# Synthesis and crystal structure of bis­(2-phthal­imido­eth­yl)ammonium chloride dihydrate

**DOI:** 10.1107/S2056989023004565

**Published:** 2023-05-26

**Authors:** Barry S. Young, Jamie L. Lee, Milan Gembicky, Jake Bailey, Gary L. N. Smith

**Affiliations:** a Department of Chemistry, San Diego Miramar College, San Diego, CA 92126, USA; b Department of Chemistry, University of California-San Diego, La Jolla, CA 92093, USA; Universidade de Sâo Paulo, Brazil

**Keywords:** crystal structure, phthalimides, π–π inter­actions, tripodal ligand

## Abstract

The title compound {systematic name: bis­[2-(1,3-dioxoisoindol-2-yl)eth­yl]aza­nium chloride dihydrate}, C_20_H_18_N_3_O_4_^+^·Cl^−^·2H_2_O, is a phthalimide-protected polyamine that was synthesized by a previous method. It was characterized by ESI–MS, ^1^H NMR, and FT–IR. Crystals were grown from a solution of H_2_O and 0.1 *M* HCl. The central nitro­gen atom is protonated and forms hydrogen bonds with the chloride ion and a water mol­ecule. The two phthalimide units make a dihedral angle of 22.07 (3)°. The crystal packing features a hydrogen-bond network, two-coordinated chloride, and off-set π–π stacking.

## Abstract 2

The title compound is a phthalimide-protected polyamine with a protonated central nitro­gen atom. The crystal packing features a hydrogen-bond network, a two-coordinated chloride ion, and off-set π–π stacking.

## 1. Chemical context

The title compound was synthesized by Frederick Mann in 1934 (Mann, 1934 ▸). It has been a key component for the synthesis of tripodal amines (Lundin *et al.*, 2004 ▸; Blackman, 2005 ▸), Schiff base macrocycles (Keypour *et al.*, 2008 ▸), MRI contrast agents of gadolinium(III) (Cheng *et al.*, 2000 ▸), and as a tricyclic host for anions (Kang *et al.*, 2010 ▸). Recently, it has also been used to functionalize graphene oxide (Ramesh & Jebasingh, 2019 ▸), build a nano-polymer dendrimer to uptake salicylic acid (Arshadi *et al.*, 2019 ▸), and construct a fluorescent ligand (Saga *et al.*, 2020 ▸). The compound itself has formed a complex with manganese as a superoxide dismutase mimetic (Piacham *et al.*, 2014 ▸). A variety of phthalimide compounds have been of inter­est because of the variety of supra­molecular inter­actions that can exist (Howell *et al.*, 2003 ▸). 
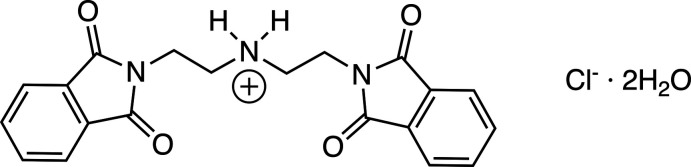


## 2. Structural commentary

**Figure 1:**
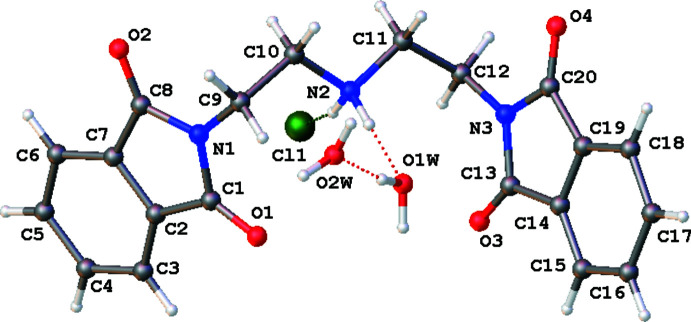
The mol­ecular structure of the title compound, showing 50% probability ellipsoids.

The mol­ecular structure of the title compound is shown in Fig. 1 ▸. The compound is a protonated polyamine with two phthalimide groups protecting the terminal nitro­gens. It crystallizes in the monoclinic space group *P*2_1_/*c*. The planes of the two phthalimide units (N1/C1–C8 and N3/C13–C20) make a dihedral angle of 22.07 (3)°. These units point in opposite directions to each other from the perspective of the central nitro­gen atom. The central tetra­hedral nitro­gen atom (NH_2_) forms hydrogen bonds with a water mol­ecule and the chloride ion.

## 3. Supra­molecular features

**Figure 2:**
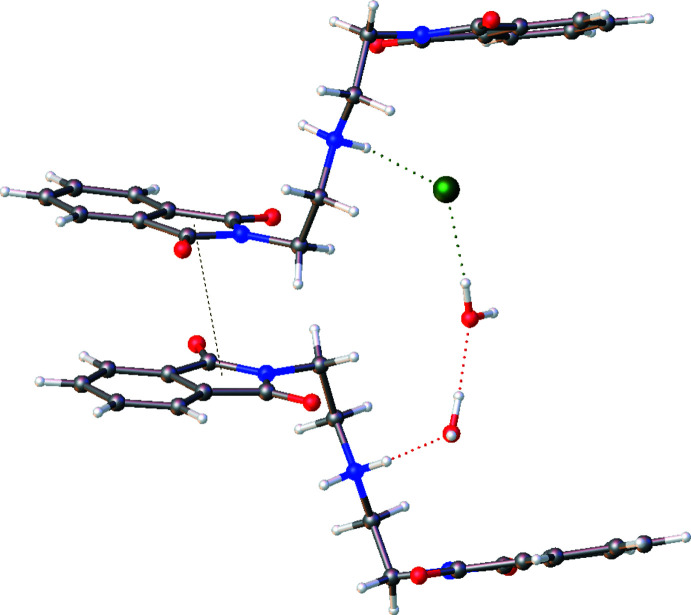
Mol­ecular packing of the title compound showing π–π inter­actions, hydrogen bonding, and chloride coordination.

The crystal structure features off-set π–π stacking between phthalimide groups running along the *b*-axis direction (Fig. 2 ▸). The *Cg* (N1/C1–C8)⋯*Cg* (N3/C13–C20) centroid–centroid distance is 4.0143 (7) Å. A hydrogen-bond network (Table 1 ▸) exists between the protonated amine (N2—H2*A*), a water mol­ecule (O1*W*), and a second water mol­ecule (O2*W*). Both water mol­ecules (O1*W*—H1*WB*, O2*W*—H2*WA*) also form hydrogen bonds with phthalimide oxygen atoms (O4, O2). The chloride ions form two hydrogen bonds with the protonated amine and a water mol­ecule.

## 4. Database survey

A search of the Cambridge Structural Database (version 5.41, update of July 2022; Groom *et al.*, 2016 ▸) for related compounds with a phthalimide unit gave 2881 hits. A search for the skeletal structure of N(CH_2_CH_2_NH_2_)_2_ resulted in 1707 hits, while the structure with protonated amines ^+^HN(CH_2_CH_2_NH_3_^+^)_2_ resulted in 182 hits. One of these structures is the triprotonated di­ethyl­enetri­amine trichloride (ETACLA01; Ilioudis *et al.*, 2000 ▸). This structure includes one chloride ion that is two-coordinate and two chlorides that are three-coordinate. A search for an amine with two phthalimide groups had 24 hits. The structure of a diphthalimidodi­ethyl­ammonium and hydrogen phthalate complex showed stabil­ization by offset π–π stacking, carbon­yl–carbonyl, and hydrogen-bonding inter­actions (REVZAT; Barrett *et al.*, 1995 ▸). Hydrogen bonding occurs within the complex unit and connects adjacent units. The offset π–π stacking between phthalimide units is characterized by C⋯C distances ranging from 3.297–3.592 Å. We have previously reported a phthalimide-protected polyamine that exhibits offset π–π stacking (Holmberg *et al.*, 2021 ▸).

## 5. Synthesis and crystallization

Following a previous protocol (Utz *et al.*, 2008 ▸), 5.0 mL (48 mmol) of di­ethyl­enetri­amine were dissolved in 50 mL of methanol. To this, 15.0 g (101 mmol) of phthalic anhydride were slowly added, which turned the solution clear and yellow. The solution was kept at 333 K with minimal fluctuations and stirred for approximately 45 min. The solution became cloudy. It was removed from heat and stirred at room temperature for 7 days. A Büchner funnel and filter paper were saturated with MeOH, and the round-bottom flask was rinsed with MeOH prior to vacuum filtration. The precipitate was a pale-yellow solid. It was rinsed four times with 25 mL of MeOH and 4 × 25 mL of acetone to give 9.609 g of the product (55% yield). Characterization results align with previous work. ESI–MS: *m*/*z* = 364.1 (*M* + H^+^), 386.1 (*M* + Na^+^). ^1^H NMR (90 MHz, CDCl_3_) δ ppm 7.75 (*m*, 8H, aromatics), 3.75 (*t*, 4H, CH_2_—N), 3.0 (*t*, 4H, CH_2_—N), 1.60 (*s*, 1H, N—H). FTIR (cm^−1^) = 3326 ν(N—H), 1698 ν(C=O). Crystals suitable for X-ray crystallography were grown by evaporation, with the compound dissolved in a solution of H_2_O and 0.1 *M* HCl.

## 6. Refinement

Crystal data, data collection and structure refinement details are summarized in Table 2 ▸. N-bound H atoms were refined with *U*_iso_(H) = 1.2*U*_eq_(N). C-bound and water H atoms were positioned geometrically (C—H = 0.96–0.99 Å, O—H = 0.87 Å) and refined as riding with *U*_iso_(H) = 1.2–1.5*U*_eq_(C, O).

## Supplementary Material

Crystal structure: contains datablock(s) I. DOI: 10.1107/S2056989023004565/ex2070sup1.cif

Structure factors: contains datablock(s) I. DOI: 10.1107/S2056989023004565/ex2070Isup2.hkl

Supporting information file. DOI: 10.1107/S2056989023004565/ex2070Isup3.mol

Supporting information file. DOI: 10.1107/S2056989023004565/ex2070Isup4.cml

CCDC reference: 2264952

Additional supporting information:  crystallographic information; 3D view; checkCIF report

## Figures and Tables

**Table 1:** Hydrogen-bond geometry (Å, °)

*D*—H⋯*A*	*D*—H	H⋯*A*	*D*⋯*A*	*D*—H⋯*A*
N2—H2*A*⋯O1*W*	0.930 (17)	1.848 (17)	2.7729 (16)	172.7 (14)
O1*W*—H1*WA*⋯O2*W*	0.87	1.88	2.7462 (15)	171
O1*W*—H1*WB*⋯O4^i^	0.87	2.05	2.9054 (14)	168
O2*W*—H2*WA*⋯O2^ii^	0.87	2.03	2.8929 (15)	172

Symmetry codes: (i) 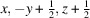 ; (ii) 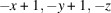 .

**Table 2:** Experimental details

Crystal data	
Chemical formula	C_20_H_18_N_3_O_4_^+^·Cl^−^·2H_2_O
*M*_r_	435.85
Crystal system, space group	Monoclinic, *P*2_1_/*c*
Temperature (K)	100
*a*, *b*, *c* (Å)	12.0401 (6), 15.4829 (7), 11.2543 (6)
β (°)	105.7191 (17)
*V* (Å^3^)	2019.52 (17)
*Z*	4
Radiation type	Mo *K*α
μ (mm^−1^)	0.23
Crystal size (mm)	0.18 × 0.18 × 0.05

Data collection	
Diffractometer	Bruker *SMART* APEXII area detector
Absorption correction	Multi-scan (*SADABS*; Krause *et al.*, 2015 ▸)
*T*_min_, *T*_max_	0.666, 0.744
No. of measured, independent and observed [*I* > 2σ(*I*)] reflections	59713, 4126, 3430
*R*_int_	0.082
(sin θ/λ)_max_ (Å^−1^)	0.625

Refinement	
*R*[*F* ^2^ > 2σ(*F* ^2^)], *wR*(*F* ^2^), *S*	0.031, 0.077, 1.03
No. of reflections	4126
No. of parameters	284
H-atom treatment	H atoms treated by a mixture of independent and constrained refinement
Δρ_max_, Δρ_min_ (e Å^−3^)	0.33, −0.23

Computer programs: *APEX4* (Bruker, 2022 ▸), *SAINT* (Bruker, 2019 ▸), *SHELXT* (Sheldrick, 2015*a* ▸), *SHELXL* (Sheldrick, 2015*b* ▸), and *OLEX2* (Dolomanov *et al.*, 2009 ▸).

## supplementary crystallographic information

### Crystal data

**Table:**

C_20_H_18_N_3_O_4_^+^·Cl^−^·2H_2_O	*F*(000) = 912
*M**_r_* = 435.85	*D*_x_ = 1.434 Mg m^−^^3^
Monoclinic, *P*2_1_/*c*	Mo *K*α radiation, λ = 0.71073 Å
*a* = 12.0401 (6) Å	Cell parameters from 8573 reflections
*b* = 15.4829 (7) Å	θ = 2.6–26.6°
*c* = 11.2543 (6) Å	µ = 0.23 mm^−^^1^
β = 105.7191 (17)°	*T* = 100 K
*V* = 2019.52 (17) Å^3^	Plate, colourless
*Z* = 4	0.18 × 0.18 × 0.05 mm

### Data collection

**Table:**

Bruker SMART APEXII area detector diffractometer	4126 independent reflections
Radiation source: Micro Focus Rotating Anode, Bruker TXS	3430 reflections with *I* > 2σ(*I*)
Double Bounce Multilayer Mirrors monochromator	*R*_int_ = 0.082
Detector resolution: 7.407 pixels mm^-1^	θ_max_ = 26.4°, θ_min_ = 2.6°
ω and φ scans	*h* = −15→15
Absorption correction: multi-scan (SADABS; Krause *et al.*, 2015)	*k* = −19→19
*T*_min_ = 0.666, *T*_max_ = 0.744	*l* = −14→14
59713 measured reflections	

### Refinement

**Table:**

Refinement on *F*^2^	Hydrogen site location: mixed
Least-squares matrix: full	H atoms treated by a mixture of independent and constrained refinement
*R*[*F*^2^ > 2σ(*F*^2^)] = 0.031	*w* = 1/[σ^2^(*F*_o_^2^) + (0.0289*P*)^2^ + 1.0135*P*] where *P* = (*F*_o_^2^ + 2*F*_c_^2^)/3
*wR*(*F*^2^) = 0.077	(Δ/σ)_max_ < 0.001
*S* = 1.03	Δρ_max_ = 0.33 e Å^−^^3^
4126 reflections	Δρ_min_ = −0.22 e Å^−^^3^
284 parameters	Extinction correction: SHELXL-2019/1 (Sheldrick 2015b), Fc^*^=kFc[1+0.001xFc^2^λ^3^/sin(2θ)]^-1/4^
0 restraints	Extinction coefficient: 0.0027 (5)
Primary atom site location: dual	

### Special details

**Table:**

Geometry. All esds (except the esd in the dihedral angle between two l.s. planes) are estimated using the full covariance matrix. The cell esds are taken into account individually in the estimation of esds in distances, angles and torsion angles; correlations between esds in cell parameters are only used when they are defined by crystal symmetry. An approximate (isotropic) treatment of cell esds is used for estimating esds involving l.s. planes.

### Fractional atomic coordinates and isotropic or equivalent isotropic displacement parameters (Å^2^)

**Table:**

	*x*	*y*	*z*	*U*_iso_*/*U*_eq_	
Cl1	0.30610 (3)	0.21490 (2)	0.23479 (3)	0.01891 (10)	
O1	0.45753 (9)	0.41672 (7)	0.37854 (9)	0.0222 (2)	
O2	0.18461 (8)	0.47720 (6)	0.01946 (9)	0.0188 (2)	
O3	0.66232 (8)	0.16482 (7)	0.41798 (9)	0.0192 (2)	
O4	0.77243 (9)	0.14778 (7)	0.06066 (9)	0.0203 (2)	
N1	0.34112 (10)	0.44489 (7)	0.18374 (11)	0.0145 (3)	
N2	0.49938 (11)	0.29689 (7)	0.14298 (11)	0.0132 (2)	
H2A	0.5674 (14)	0.3152 (10)	0.1984 (15)	0.016*	
H2B	0.4486 (14)	0.2814 (10)	0.1872 (15)	0.016*	
N3	0.68861 (10)	0.15627 (7)	0.22242 (11)	0.0144 (3)	
C1	0.36218 (13)	0.42849 (9)	0.31025 (13)	0.0168 (3)	
C2	0.24799 (13)	0.43156 (9)	0.33647 (13)	0.0172 (3)	
C3	0.21870 (14)	0.41977 (10)	0.44585 (14)	0.0232 (3)	
H3	0.275327	0.407303	0.520828	0.028*	
C4	0.10228 (15)	0.42703 (10)	0.44106 (15)	0.0264 (4)	
H4	0.078868	0.418693	0.514353	0.032*	
C5	0.01953 (14)	0.44615 (10)	0.33171 (16)	0.0254 (4)	
H5	−0.059106	0.450841	0.331866	0.030*	
C6	0.04974 (13)	0.45861 (9)	0.22169 (15)	0.0208 (3)	
H6	−0.006455	0.471957	0.146751	0.025*	
C7	0.16529 (13)	0.45060 (9)	0.22689 (13)	0.0165 (3)	
C8	0.22447 (12)	0.45952 (9)	0.12767 (13)	0.0146 (3)	
C9	0.43146 (12)	0.45125 (9)	0.12044 (13)	0.0163 (3)	
H9A	0.504935	0.466733	0.181225	0.020*	
H9B	0.411828	0.498362	0.058814	0.020*	
C10	0.44862 (12)	0.36834 (9)	0.05565 (13)	0.0160 (3)	
H10A	0.373203	0.349242	0.001902	0.019*	
H10B	0.500022	0.380153	0.002199	0.019*	
C11	0.51826 (12)	0.21954 (9)	0.07135 (13)	0.0147 (3)	
H11A	0.571103	0.235377	0.021083	0.018*	
H11B	0.443688	0.202189	0.014086	0.018*	
C12	0.56858 (12)	0.14316 (9)	0.15283 (13)	0.0159 (3)	
H12A	0.521779	0.132557	0.211393	0.019*	
H12B	0.563460	0.091081	0.100582	0.019*	
C13	0.72536 (12)	0.16741 (9)	0.35046 (13)	0.0144 (3)	
C14	0.85216 (12)	0.18028 (9)	0.38127 (13)	0.0146 (3)	
C15	0.93036 (12)	0.19494 (9)	0.49353 (13)	0.0173 (3)	
H15	0.907043	0.196796	0.567657	0.021*	
C16	1.04548 (13)	0.20700 (9)	0.49417 (14)	0.0196 (3)	
H16	1.101625	0.218356	0.569922	0.024*	
C17	1.07905 (12)	0.20260 (9)	0.38529 (14)	0.0192 (3)	
H17	1.157746	0.211544	0.387946	0.023*	
C18	0.99976 (12)	0.18540 (9)	0.27284 (14)	0.0176 (3)	
H18	1.023044	0.180774	0.198902	0.021*	
C19	0.88563 (12)	0.17528 (9)	0.27268 (13)	0.0152 (3)	
C20	0.78104 (12)	0.15836 (9)	0.16934 (13)	0.0154 (3)	
O1W	0.70481 (9)	0.36110 (7)	0.29278 (9)	0.0200 (2)	
H1WA	0.715322	0.413414	0.269788	0.030*	
H1WB	0.717563	0.364549	0.372498	0.030*	
O2W	0.72498 (11)	0.52060 (7)	0.19391 (11)	0.0287 (3)	
H2WA	0.758477	0.523958	0.134596	0.043*	
H2WB	0.720782	0.573778	0.217062	0.043*	

### Atomic displacement parameters (Å^2^)

**Table:**

	*U*^11^	*U*^22^	*U*^33^	*U*^12^	*U*^13^	*U*^23^
Cl1	0.01688 (18)	0.02079 (19)	0.02101 (19)	−0.00147 (14)	0.00846 (14)	0.00137 (14)
O1	0.0220 (6)	0.0210 (6)	0.0211 (6)	0.0023 (4)	0.0014 (5)	0.0030 (4)
O2	0.0189 (5)	0.0207 (5)	0.0168 (5)	0.0013 (4)	0.0049 (4)	0.0020 (4)
O3	0.0178 (5)	0.0221 (6)	0.0205 (5)	0.0004 (4)	0.0099 (4)	−0.0007 (4)
O4	0.0240 (6)	0.0228 (6)	0.0151 (5)	0.0011 (4)	0.0071 (4)	−0.0004 (4)
N1	0.0146 (6)	0.0143 (6)	0.0160 (6)	0.0023 (5)	0.0062 (5)	0.0014 (5)
N2	0.0133 (6)	0.0129 (6)	0.0139 (6)	0.0002 (5)	0.0048 (5)	0.0006 (5)
N3	0.0139 (6)	0.0141 (6)	0.0157 (6)	0.0007 (4)	0.0050 (5)	−0.0005 (5)
C1	0.0221 (8)	0.0103 (7)	0.0180 (7)	0.0016 (6)	0.0053 (6)	0.0001 (5)
C2	0.0220 (7)	0.0110 (7)	0.0197 (7)	0.0016 (6)	0.0079 (6)	−0.0008 (5)
C3	0.0337 (9)	0.0183 (8)	0.0201 (8)	0.0053 (6)	0.0116 (7)	0.0020 (6)
C4	0.0382 (10)	0.0219 (8)	0.0270 (9)	0.0029 (7)	0.0222 (8)	0.0011 (7)
C5	0.0251 (8)	0.0205 (8)	0.0372 (9)	0.0003 (6)	0.0198 (7)	−0.0021 (7)
C6	0.0194 (8)	0.0180 (8)	0.0266 (8)	0.0017 (6)	0.0092 (6)	−0.0016 (6)
C7	0.0202 (7)	0.0115 (7)	0.0198 (7)	0.0007 (5)	0.0087 (6)	−0.0006 (6)
C8	0.0169 (7)	0.0102 (6)	0.0170 (7)	0.0006 (5)	0.0052 (6)	−0.0016 (5)
C9	0.0147 (7)	0.0146 (7)	0.0218 (8)	0.0007 (5)	0.0085 (6)	0.0024 (6)
C10	0.0179 (7)	0.0151 (7)	0.0160 (7)	0.0033 (6)	0.0063 (6)	0.0043 (6)
C11	0.0154 (7)	0.0136 (7)	0.0154 (7)	0.0012 (5)	0.0046 (6)	−0.0014 (5)
C12	0.0141 (7)	0.0141 (7)	0.0190 (7)	−0.0003 (5)	0.0036 (6)	−0.0003 (6)
C13	0.0179 (7)	0.0095 (7)	0.0168 (7)	0.0021 (5)	0.0063 (6)	0.0004 (5)
C14	0.0154 (7)	0.0108 (7)	0.0187 (7)	0.0024 (5)	0.0062 (6)	0.0009 (5)
C15	0.0196 (7)	0.0162 (7)	0.0169 (7)	0.0031 (6)	0.0063 (6)	0.0001 (6)
C16	0.0177 (7)	0.0179 (8)	0.0216 (8)	0.0026 (6)	0.0023 (6)	−0.0003 (6)
C17	0.0136 (7)	0.0177 (7)	0.0270 (8)	0.0022 (6)	0.0067 (6)	0.0027 (6)
C18	0.0185 (7)	0.0171 (7)	0.0199 (7)	0.0030 (6)	0.0100 (6)	0.0031 (6)
C19	0.0180 (7)	0.0115 (7)	0.0167 (7)	0.0021 (5)	0.0058 (6)	0.0018 (5)
C20	0.0193 (7)	0.0102 (7)	0.0185 (8)	0.0021 (5)	0.0080 (6)	0.0017 (5)
O1W	0.0225 (6)	0.0178 (5)	0.0183 (5)	−0.0029 (4)	0.0029 (4)	0.0011 (4)
O2W	0.0453 (7)	0.0196 (6)	0.0285 (6)	0.0027 (5)	0.0225 (6)	0.0041 (5)

### Geometric parameters (Å, º)

**Table:**

O1—C1	1.2099 (17)	C9—H9A	0.9900
O2—C8	1.2131 (17)	C9—H9B	0.9900
O3—C13	1.2115 (17)	C9—C10	1.5179 (19)
O4—C20	1.2103 (17)	C10—H10A	0.9900
N1—C1	1.4001 (18)	C10—H10B	0.9900
N1—C8	1.3936 (18)	C11—H11A	0.9900
N1—C9	1.4561 (18)	C11—H11B	0.9900
N2—H2A	0.930 (17)	C11—C12	1.5184 (19)
N2—H2B	0.919 (17)	C12—H12A	0.9900
N2—C10	1.4959 (17)	C12—H12B	0.9900
N2—C11	1.4950 (17)	C13—C14	1.4845 (19)
N3—C12	1.4594 (18)	C14—C15	1.375 (2)
N3—C13	1.3988 (18)	C14—C19	1.389 (2)
N3—C20	1.3988 (18)	C15—H15	0.9500
C1—C2	1.483 (2)	C15—C16	1.397 (2)
C2—C3	1.381 (2)	C16—H16	0.9500
C2—C7	1.391 (2)	C16—C17	1.392 (2)
C3—H3	0.9500	C17—H17	0.9500
C3—C4	1.393 (2)	C17—C18	1.389 (2)
C4—H4	0.9500	C18—H18	0.9500
C4—C5	1.389 (2)	C18—C19	1.382 (2)
C5—H5	0.9500	C19—C20	1.488 (2)
C5—C6	1.395 (2)	O1W—H1WA	0.8699
C6—H6	0.9500	O1W—H1WB	0.8699
C6—C7	1.382 (2)	O2W—H2WA	0.8700
C7—C8	1.485 (2)	O2W—H2WB	0.8692
			
C1—N1—C9	123.81 (12)	N2—C10—H10A	108.9
C8—N1—C1	111.88 (11)	N2—C10—H10B	108.9
C8—N1—C9	124.21 (12)	C9—C10—H10A	108.9
H2A—N2—H2B	108.2 (13)	C9—C10—H10B	108.9
C10—N2—H2A	110.1 (10)	H10A—C10—H10B	107.7
C10—N2—H2B	109.6 (10)	N2—C11—H11A	109.0
C11—N2—H2A	111.8 (10)	N2—C11—H11B	109.0
C11—N2—H2B	107.7 (10)	N2—C11—C12	113.10 (11)
C11—N2—C10	109.44 (11)	H11A—C11—H11B	107.8
C13—N3—C12	124.12 (11)	C12—C11—H11A	109.0
C13—N3—C20	111.77 (11)	C12—C11—H11B	109.0
C20—N3—C12	124.11 (12)	N3—C12—C11	113.01 (11)
O1—C1—N1	123.53 (13)	N3—C12—H12A	109.0
O1—C1—C2	130.53 (14)	N3—C12—H12B	109.0
N1—C1—C2	105.92 (12)	C11—C12—H12A	109.0
C3—C2—C1	130.34 (14)	C11—C12—H12B	109.0
C3—C2—C7	121.60 (14)	H12A—C12—H12B	107.8
C7—C2—C1	108.06 (12)	O3—C13—N3	124.40 (13)
C2—C3—H3	121.6	O3—C13—C14	129.59 (13)
C2—C3—C4	116.88 (15)	N3—C13—C14	105.99 (11)
C4—C3—H3	121.6	C15—C14—C13	129.93 (13)
C3—C4—H4	119.2	C15—C14—C19	121.88 (13)
C5—C4—C3	121.61 (14)	C19—C14—C13	108.19 (12)
C5—C4—H4	119.2	C14—C15—H15	121.4
C4—C5—H5	119.4	C14—C15—C16	117.29 (13)
C4—C5—C6	121.25 (15)	C16—C15—H15	121.4
C6—C5—H5	119.4	C15—C16—H16	119.6
C5—C6—H6	121.6	C17—C16—C15	120.89 (14)
C7—C6—C5	116.84 (15)	C17—C16—H16	119.6
C7—C6—H6	121.6	C16—C17—H17	119.3
C2—C7—C8	108.21 (12)	C18—C17—C16	121.30 (13)
C6—C7—C2	121.82 (14)	C18—C17—H17	119.3
C6—C7—C8	129.97 (14)	C17—C18—H18	121.3
O2—C8—N1	124.57 (13)	C19—C18—C17	117.42 (13)
O2—C8—C7	129.49 (13)	C19—C18—H18	121.3
N1—C8—C7	105.93 (12)	C14—C19—C20	108.16 (12)
N1—C9—H9A	108.9	C18—C19—C14	121.18 (13)
N1—C9—H9B	108.9	C18—C19—C20	130.65 (13)
N1—C9—C10	113.25 (11)	O4—C20—N3	124.62 (13)
H9A—C9—H9B	107.7	O4—C20—C19	129.53 (13)
C10—C9—H9A	108.9	N3—C20—C19	105.85 (11)
C10—C9—H9B	108.9	H1WA—O1W—H1WB	104.5
N2—C10—C9	113.22 (11)	H2WA—O2W—H2WB	104.5
			
O1—C1—C2—C3	−1.7 (3)	C9—N1—C1—O1	−1.2 (2)
O1—C1—C2—C7	177.56 (15)	C9—N1—C1—C2	177.37 (12)
O3—C13—C14—C15	−2.0 (2)	C9—N1—C8—O2	1.8 (2)
O3—C13—C14—C19	177.98 (14)	C9—N1—C8—C7	−177.10 (12)
N1—C1—C2—C3	179.83 (14)	C10—N2—C11—C12	−179.36 (11)
N1—C1—C2—C7	−0.86 (15)	C11—N2—C10—C9	−177.34 (11)
N1—C9—C10—N2	−69.13 (15)	C12—N3—C13—O3	2.3 (2)
N2—C11—C12—N3	−70.61 (15)	C12—N3—C13—C14	−178.85 (12)
N3—C13—C14—C15	179.24 (14)	C12—N3—C20—O4	−1.6 (2)
N3—C13—C14—C19	−0.76 (15)	C12—N3—C20—C19	178.60 (12)
C1—N1—C8—O2	178.13 (13)	C13—N3—C12—C11	110.75 (14)
C1—N1—C8—C7	−0.76 (15)	C13—N3—C20—O4	177.76 (13)
C1—N1—C9—C10	97.89 (15)	C13—N3—C20—C19	−1.99 (15)
C1—C2—C3—C4	179.82 (14)	C13—C14—C15—C16	−178.35 (13)
C1—C2—C7—C6	−179.45 (13)	C13—C14—C19—C18	179.61 (13)
C1—C2—C7—C8	0.42 (15)	C13—C14—C19—C20	−0.42 (15)
C2—C3—C4—C5	−0.7 (2)	C14—C15—C16—C17	−1.2 (2)
C2—C7—C8—O2	−178.64 (14)	C14—C19—C20—O4	−178.29 (14)
C2—C7—C8—N1	0.18 (15)	C14—C19—C20—N3	1.45 (15)
C3—C2—C7—C6	−0.1 (2)	C15—C14—C19—C18	−0.4 (2)
C3—C2—C7—C8	179.80 (13)	C15—C14—C19—C20	179.58 (13)
C3—C4—C5—C6	0.2 (2)	C15—C16—C17—C18	−0.6 (2)
C4—C5—C6—C7	0.3 (2)	C16—C17—C18—C19	1.8 (2)
C5—C6—C7—C2	−0.4 (2)	C17—C18—C19—C14	−1.4 (2)
C5—C6—C7—C8	179.78 (14)	C17—C18—C19—C20	178.68 (14)
C6—C7—C8—O2	1.2 (3)	C18—C19—C20—O4	1.7 (3)
C6—C7—C8—N1	−179.96 (14)	C18—C19—C20—N3	−178.59 (14)
C7—C2—C3—C4	0.6 (2)	C19—C14—C15—C16	1.6 (2)
C8—N1—C1—O1	−177.56 (13)	C20—N3—C12—C11	−69.92 (16)
C8—N1—C1—C2	1.01 (15)	C20—N3—C13—O3	−177.07 (13)
C8—N1—C9—C10	−86.20 (16)	C20—N3—C13—C14	1.75 (15)

### Hydrogen-bond geometry (Å, º)

**Table:**

*D*—H···*A*	*D*—H	H···*A*	*D*···*A*	*D*—H···*A*
N2—H2*A*···O1*W*	0.930 (17)	1.848 (17)	2.7729 (16)	172.7 (14)
O1*W*—H1*WA*···O2*W*	0.87	1.88	2.7462 (15)	171
O1*W*—H1*WB*···O4^i^	0.87	2.05	2.9054 (14)	168
O2*W*—H2*WA*···O2^ii^	0.87	2.03	2.8929 (15)	172

Symmetry codes: (i) *x*, −*y*+1/2, *z*+1/2; (ii) −*x*+1, −*y*+1, −*z*.
